# Lost in the Crowd: How Does Human 8-Oxoguanine DNA Glycosylase 1 (OGG1) Find 8-Oxoguanine in the Genome?

**DOI:** 10.3390/ijms21218360

**Published:** 2020-11-07

**Authors:** Ostiane D’Augustin, Sébastien Huet, Anna Campalans, Juan Pablo Radicella

**Affiliations:** 1 Institute of Cellular and Molecular Radiobiology, Institut de Biologie François Jacob, CEA, Université Paris-Saclay, Université de Paris, 18 route du Panorama, F-92265 Fontenay-aux-Roses, France; ostiane.daugustin@univ-rennes1.fr; 2 Univ Rennes, CNRS, IGDR (Institut de génétique et développement de Rennes)-UMR 6290, BIOSIT-UMS3480, F-35000 Rennes, France; 3 Institut Universitaire de France, F-75000 Paris, France

**Keywords:** OGG1, 8-oxoG, DNA repair, base excision repair, search mechanism

## Abstract

The most frequent DNA lesion resulting from an oxidative stress is 7,8-dihydro-8-oxoguanine (8-oxoG). 8-oxoG is a premutagenic base modification due to its capacity to pair with adenine. Thus, the repair of 8-oxoG is critical for the preservation of the genetic information. Nowadays, 8-oxoG is also considered as an oxidative stress-sensor with a putative role in transcription regulation. In mammalian cells, the modified base is excised by the 8-oxoguanine DNA glycosylase (OGG1), initiating the base excision repair (BER) pathway. OGG1 confronts the massive challenge that is finding rare occurrences of 8-oxoG among a million-fold excess of normal guanines. Here, we review the current knowledge on the search and discrimination mechanisms employed by OGG1 to find its substrate in the genome. While there is considerable data from in vitro experiments, much less is known on how OGG1 is recruited to chromatin and scans the genome within the cellular nucleus. Based on what is known of the strategies used by proteins searching for rare genomic targets, we discuss the possible scenarios allowing the efficient detection of 8-oxoG by OGG1.

## 1. 8-Oxoguanine: Biological Relevance and Repair

### 1.1. Biological Prominence and Consequences of 8-Oxoguanine

Reactive oxygen species (ROS) arise within the cell as a consequence of the cellular metabolism as well as of the exposure to environmental factors. While ROS are required for the normal functioning of the cell, an intracellular excess of ROS leads to oxidative stress resulting in the abnormal oxidation of biological macromolecules. In the case of DNA, ROS can induce a plethora of chemical modifications, including strand breaks and oxidized bases. Amongst the latter, 7,8-dihydro-8-oxoguanine (8-oxoG), an oxidized form of guanine, is the most abundant [1,2]. Indeed, in human cells 8-oxoG is present at a steady state level of two to three residues per 10^6^ guanines [3,4]. Its predominance is likely due to guanine having the lowest oxidative potential among all DNA (and RNA) bases [5,6]. Guanine can be oxidized directly or upon electron transfer from one base to another over distances up to 37 Å due to π-stacking interactions between the bases, the 5′-G of a 5′-GG-3′ doublet being the most likely to be oxidized [7].

Accumulation of oxidative base damage in genomic DNA and deficiency in its repair, have been associated with several pathologies and ageing [8,9]. While these effects mostly originate from mutations consecutive to base oxidation, 8-oxoG was also reported to interfere with telomere replication leading to genomic instability and ageing-related diseases [10,11,12,13,14]. Oxidation of mitochondrial DNA has also been implicated in neurodegenerative diseases and ageing. Confirming the deleterious consequences of 8-oxoG accumulation in DNA, mutations affecting the genes coding for the enzymes involved in the repair of this lesion are linked to several human diseases [15].

**Figure 1:**
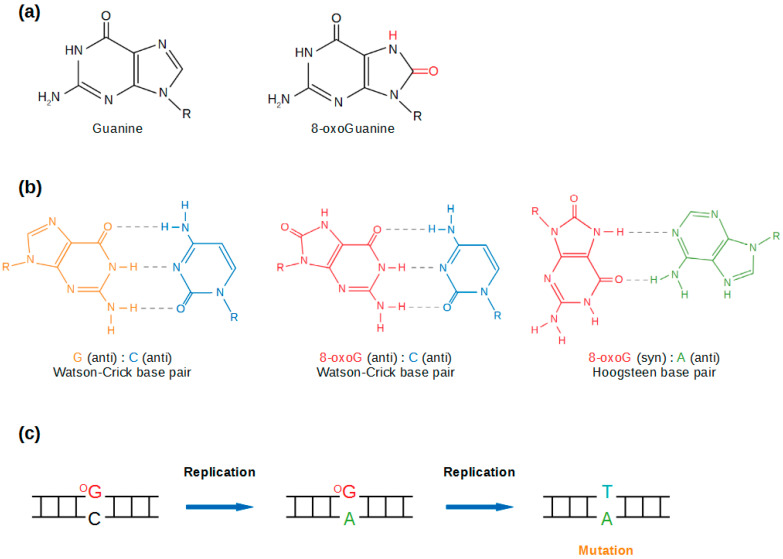
8-oxoG is a premutagenic lesion. (**a**) 8-oxoG and G. Differences are represented in red. (**b**) G:C, 8-oxoG:C and 8-oxoG:A base pairs. (**c**) Fixation of a transversion due to the presence of 8-oxoG.

8-oxoG only differs from normal guanine by two atoms: C8 and N7, harbouring an oxygen instead of a hydrogen, and a hydrogen instead of an electron pair, respectively (Figure 1a). While 8-oxoG affects the thermodynamic stability of the duplex [16,17], structural studies showed that its presence induces little or no distortion of the DNA helix, yielding a normal Watson–Crick base-paring arrangement [18,19,20]. As a consequence, 8-oxoG does not block DNA replication, although it may decrease the rate of DNA synthesis or induce pausing of the polymerase [21]. Nor does it constitute a permanent barrier to transcription [22]. Yet, the capacity of 8-oxoG to form a Hoogsteen base pair with an adenine through an *anti-syn* conformation (Figure 1b) [19,23] leads to the frequent incorporation of adenine opposite to 8-oxoG during DNA synthesis [24,25]. Upon further replication, a G:C to T:A transversion is fixed, making of 8-oxoG a highly pre-mutagenic lesion [26] (Figure 1c). Similarly, the 8-oxoG:A Hoogsteen base pair can occur during transcription, resulting in mutant RNAs [27].

### 1.2. 8-oxoG Repair in Mammalian Cells

In cells, the removal of non-canonical or damaged bases from DNA is achieved mainly by the base excision repair (BER) pathway. This pathway has been extensively reviewed over time [28,29,30]. The initial step in the repair is the recognition of the modified base and cleavage of the N-glycosydic bond by a specific DNA glycosylase to generate an abasic (AP) site. The AP site is processed by the downstream enzymes of the pathway.

The discovery and characterization of the yeast 8-oxoG DNA glycosylase [31,32] allowed the identification of the mammalian orthologue (OGG1) [33,34,35,36,37,38,39]. Like the yeast enzyme, mammalian OGG1 has a strong preference for the excision of 8-oxoG paired to a cytosine, rather than to other bases. Cleavage of the 8-oxoG N-glycosydic bond by OGG1 leaves an AP site. While OGG1 can act in vitro as a bifunctional DNA glycosylase, the β-elimination cleavage of the AP site seems to be uncoupled from the DNA glycosylase activity [40,41,42].

Although other DNA glycosylases as well as a reconstituted nucleotide excision repair (NER) system are capable of excising 8-oxoG from oligonucleotides, in all cases their activity is very low compared to that of OGG1 and the relevance for their role in preventing 8-oxoG-induced mutagenesis remains unproven [15,43]. Furthermore, OGG1 appears unable to excise 8-oxoG in the context of tandem lesions, suggesting that alternative DNA glycosylases or repair systems other than BER (i.e., NER) could handle 8-oxoG in those contexts [44].

## 2. How Does OGG1 Find 8-oxoG Amongst a Million-Fold Excess of Gs?

The molecular mechanism of 8-oxoG excision by DNA glycosylases has been thoroughly explored by biochemical and structural approaches [15,45]. Here, we will focus on the search and recognition of 8-oxoG by OGG1. As mentioned above, the presence of 8-oxoG does not induce helix distortion, neither does it create replication or transcription blockage, raising a set of challenges for OGG1 in order to efficiently locate the oxidized guanine against a high background of undamaged bases.

### 2.1. 8-oxoG Recognition by OGG1 on Naked DNA

The mechanism of lesion recognition by OGG1 has received extensive attention, mostly through the study of the structure of the protein and some mutant derivatives in the presence of DNA harbouring an 8-oxoG residue. The initial studies led to propose a mechanism by which the enzyme facilitates the extrusion (flip-out) of the damaged base from the interior of the DNA helix due to a disruption of the base pairing induced by local DNA bending [46]. In this model, the specificity for 8-oxoG would be ensured by the fact that 8-oxoG:C pairs show increased flexibility and a reduced base extrusion energy barrier compared to G-C pairs [47]. However, OGG1 interrogates undamaged DNA by inducing drastic kinks [48] also extruding normal guanine residues into a so-called “exosite” in the protein [49,50,51]. Molecular dynamics simulations suggest that DNA deformation provides several gating steps for the recognition of the modified base [52]. Consistently, OGG1 conformation seems to evolve during the different steps, from the interrogation of undamaged DNA (interrogation complex), the encounter of the 8-oxoG and its extrusion (encounter complex) and then final recognition (lesion recognition complex) [53]. These observations indicate that the discrimination between a normal G and an 8-oxoG must occur after the flipping out of the base. As mentioned earlier, G and 8-oxoG only differ by the addition of a hydrogen at position N7 and an oxygen at position 8 (Figure 1). The structure of the active site mutant OGG1(K249Q) with an 8-oxoG in its catalytic pocket suggested that there is no direct contact of the enzyme with the 8-oxo group. Therefore, the formation of a hydrogen bond between N7H and Gly42 was proposed to favour the retention of the damaged base, while its absence in the case of a normal guanine would result in the rejection of the base from the exosite. More recent work suggests that Lys249 is indispensable for 8-oxoG final alignment in the catalytic pocket, probably through interaction with O8 in the oxidized base [40]. Molecular dynamic simulations together with pre-steady-state fluorescence kinetics support the role of residues Lys249 and Asp268 in the correct positioning of 8-oxoG suitable for the catalytic hydrolysis of the N-glycosidic bond [54]. Specific contacts with the estranged C opposite the lesion provide a mean for OGG1 to discriminate against the processing of 8-oxoG paired with other bases [46].

**Figure 2:**
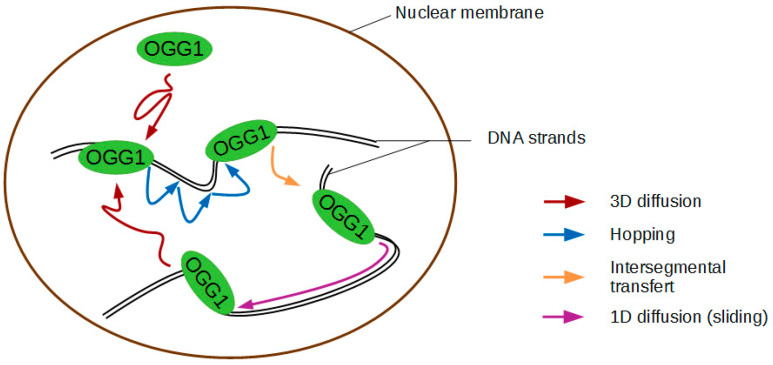
Proposed OGG1 diffusion modes.

The paragraph above described the requirements for OGG1 recognition of 8-oxoG once the enzyme is bound to the DNA at the site of the lesion. However, before reaching that point OGG1 has to scan the DNA. In the case of DNA glycosylases, the mechanism invoked is that of facilitated diffusion by which DNA binding proteins stochastically bind and diffuse along DNA to locate their target [55]. DNA glycosylases do not consume biochemical energy to drive their diffusion, their scanning of the DNA is therefore thermally activated and unbiased in its direction. For 1D diffusion along DNA, two options exist: sliding, by which the protein undergoes diffusion while remaining bound to the DNA, and hopping, referring to a repeated dissociation and rebinding at a new nearby location (Figure 2) [55,56]. Early single molecule experiments on stretched DNA (1D search) suggested that OGG1 slides in persistent contact with DNA [50]. They showed that it does so with a diffusion constant approaching the theoretical upper limit for one-dimensional diffusion, allowing OGG1 to scan up to 1 kb in 0.1 s at nearly physiological pH and salt concentration. Single-molecule diffusion data indicated that this rapid sliding is coupled to rotation to keep the DNA binding interface of the protein in constant contact with DNA [57]. More recent experiments using higher sampling rates [58] confirmed that the main mode of diffusion for OGG1 on DNA is helical sliding. Single molecule studies on the bacterial Nth, Nei, Fpg, and MutY have recently showed that these DNA glycosylases involved in the repair of oxidative DNA damage also scan for their cognate lesions at rates consistent with random rotational diffusion along the DNA backbone [59,60]. Interestingly, the values of activation energy obtained using instantaneous diffusion rate results are consistent with OGG1 examining flipped-out bases one by one. While the initial in vitro data suggested OGG1 sliding along DNA could occur over length scales of hundreds of bases, in cells the enzyme will be most probably forced to dissociate once it reaches inaccessible stretches of DNA occluded by bound proteins, thus alternating hopping and sliding modes. This scenario is further supported by observations using the high-resolution molecular clock method showing that OGG1 sliding on DNA actually occurs by microscopic 2D and 3D hopping steps [61]. The behaviour of the protein will thus depend on the degree of DNA compaction, the concentration of the enzyme and the molecular crowding. The relatively short mean sliding length calculated [50,61] seems compatible with searching short segments of DNA accessible within the cell. This Brownian target search provides high redundancy in the search for the modified base. Interestingly, three DNA glycosylases required for the removal of oxidised bases in *E. coli* were shown to scan along the DNA in a manner similar to that of OGG1. All moved randomly in both directions and scanned repeatedly over a single area of DNA with high redundancy [62].

### 2.2. Impact of the Nucleosome on 8-oxoG Excision by OGG1

DNA organisation in chromatin represents a challenge for DNA repair enzymes [63,64]. This can be particularly true in the case of OGG1 search for its cognate lesion, 8-oxoG, that does not create a significant distortion of the helix, therefore unlikely to affect the chromatin context in its surroundings. The high level of compaction of the genome and the presence of proteins bound to the DNA would act as barriers for the scanning by OGG1.

The first level of chromatin organisation is the nucleosome core particle in which 147 base pairs of DNA are wrapped around a histone octamer. Compared to the naked DNA substrate, the excision activity of the two major human uracil DNA glycosylases, UNG2 and SMUG1, is decreased by 3- to 9-fold when the uracil-containing DNA is wrapped around a nucleosome core particle [65]. While in this study the reduction of uracil removal was independent of the position of the lesion within the core particle, a later report showed that the inhibition of the glycosylase was more pronounced when the uracil faced towards the histones [66].

Similarly, while OGG1-mediated excision of an 8-oxoG embedded in a reconstituted nucleosome can be facilitated by spontaneous and transient unwrapping of DNA from the histones [67], the efficiency is reduced when compared to that on a naked DNA substrate [68]. Incubation of the same nucleosome substrate with the chromatin remodeller SWI/SNF relieved this inhibition. The same authors showed that removal of 8-oxoG present within the linker DNA is impaired by the presence of histone H1 [69]. Based on these observations, it was suggested that *in vivo* chromatin remodellers or histone chaperones are required for efficient 8-oxoG repair in the cell. Nevertheless, in such in vitro reconstituted chromatin substrates, any biochemical activity disturbing the nucleosome structure might increase the exposure of the lesion, thus promoting its excision. This is not necessarily true in the context of a fully folded genome and therefore further experiments are required to prove the need for these factors for efficient 8-oxoG removal in living cells.

### 2.3. Mechanisms Triggering the Recruitment of OGG1 to 8-oxoG Lesions in the Nuclear Context

**Figure 3:**
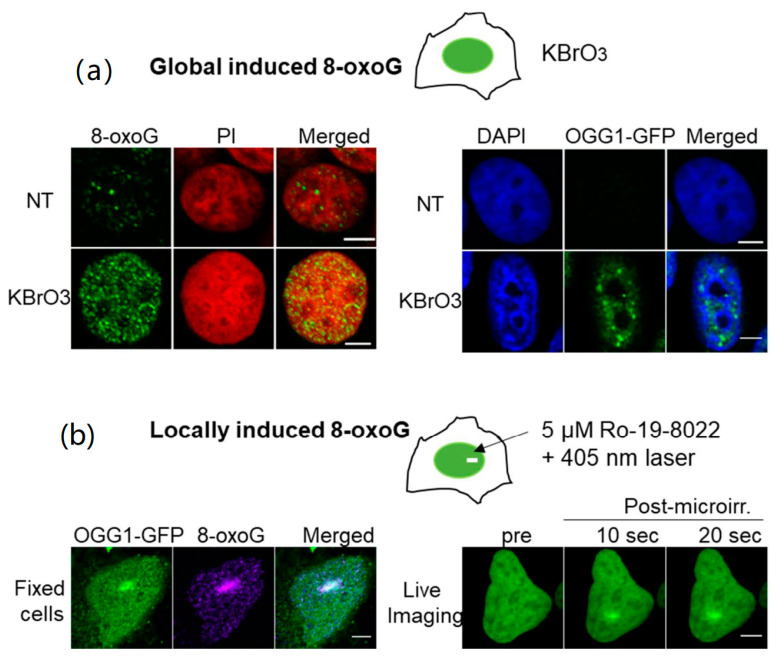
Induction of 8-oxoG and recruitment of OGG1-GFP to chromatin. (**a**) Exposure of cells to KBrO_3_ results in a global induction of 8-oxoG in the nuclear genome leading to recruitment of OGG1 to chromatin. Left panel: 8-oxoG was detected using a specific antibody against the lesion (green) and DNA was stained with PI (red). Right panel: The fusion of OGG1 to the fluorescent protein GFP (green), allows following the distribution of the protein in untreated cells or cells exposed to KBrO_3_. The soluble fraction was extracted before imaging to detect the association of the protein with chromatin. Nuclear DNA was stained with DAPI (blue). Scale bars: 5 μm. (**b**) Recruitment of OGG1-GFP to 8-oxoG induced by laser micro-irradiation. Left panel: The antibody against 8-oxoG (magenta) reveals the formation of the lesion at the same regions where OGG1-GFP (green) was recruited. Right panel: Recruitment of OGG1-GFP in real time by live-cell imaging. Scale bars: 5 μm.

Another level of complexity is reached when we consider that OGG1 has to find the 8-oxoG in the extremely dense nuclear environment. In this context, OGG1 has to deal not only with chromatin architecture, but also with a plethora of other proteins involved in replication, transcription and DNA repair that also explore the nuclear space to find their targets. In unchallenged cells, OGG1 is mostly present in the nucleoplasm soluble fraction and is extremely dynamic [70,71,72]. Our experiments showed that upon treatment with oxidizing agents such as KBrO_3_ or H_2_O_2_, which generate 8-oxoG in the cellular DNA, a fraction of OGG1 becomes insoluble due to its recruitment or retention at the chromatin (Figure 3a) [70]. This results in a decrease in OGG1 nuclear mobility assessed by fluorescence redistribution after photoperturbation (FRAP) experiments [71,72,73]. Experiments performed in our laboratory demonstrated that OGG1 is recruited in a PARP independent manner within a few seconds to the region of damage after local induction of 8-oxoG lesions by laser microirradiation (Figure 3b) [69,71,74]. The time of retention of OGG1 in the chromatin fraction correlates with the repair kinetics of the 8-oxoG [70,71]. Surprisingly, the OGG1(F319A) mutant that is deficient in the recognition of 8-oxoG, is also recruited to the chromatin fraction in KBrO_3_ treated cells [70]. This intriguing result suggests that OGG1 recruitment to the sites of damage is not triggered by the detection of the modified base by the glycosylase. In the sections below, we will describe the additional mechanisms that are proposed to contribute to the efficient accumulation of OGG1 at the sites of oxidative DNA damage.

#### 2.3.1. Efficient Recruitment of OGG1 to Chromatin Is Promoted by Several Cofactors

One of the earliest proteins recruited upon DNA damage induction is poly-ADP-ribose (PAR) polymerase 1 (PARP1) which signals DNA damage by adding PAR chains to the surroundings proteins, mostly histones and PARP1 itself, increasing chromatin accessibility [75,76]. Although several reports suggest a role of PARP1 in BER, it seems to be mostly linked to the processing of intermediates of the pathway and not to the initiation step mediated by DNA glycosylases. Indeed, it was shown that PARylation is not required for recruitment of OGG1 to the chromatin fraction upon oxidative stress or laser induced 8-oxoG [71].

Proteins from the NER pathway specialized in the repair of bulky DNA lesions facilitate the repair of 8-oxoG and other oxidised bases. In most cases, it is by the stimulation of the BER enzymes but it has also been suggested that they could participate in chromatin remodelling and thus facilitate the access of other repair factors [77]. UV-DDB1, a NER damage sensor has been implicated in the BER of 8-oxoG. UV-DDB1 is recruited to 8-oxoG induced at telomeres where it interacts and stimulates the enzymatic activity of OGG1, MYH, and APE1 (which cleave adenine paired to 8-oxoG, and processes the AP site, respectively) and may thus be involved in the orchestration of the first steps of BER [78]. However, because UV-DDB1 affinity for an 8-oxoG:C pair is much lower than that of OGG1 [79], it is unlikely that it can act as the damage recognition for the oxidized guanine. Beyond the role of UV-DDB1 in BER, a larger cooperation and interplay between BER and NER has been well documented [80]. XPC was the first NER protein shown to have a role in BER of 8-oxoG via its ability to interact with OGG1 and stimulate its activity [81]. Later on, XPC and CSB were shown to be recruited to laser micro-irradiation induced 8-oxoG [69,82] and a reduced efficiency in the removal of 8-oxoG was observed in cells deficient in CSB, XPA, and XPC, all three NER proteins [83].

Another indication of the possible crosstalk between 8-oxoG BER and other DNA repair mechanisms is the interaction between OGG1 and hSSB1, a protein involved in the repair of double strand breaks by homologous recombination. hSSB1 re-localises to the chromatin fraction upon cell exposure to H_2_O_2_ and is required for the retention of OGG1 on chromatin and the excision of 8-oxoG [84]. In vitro, hSSB1 can interact with a dsDNA containing an 8-oxoG and can mediate the binding of the OGG1(F319A) mutant, that, as mentioned before, has no affinity for the lesion. Those results could explain the recruitment of the OGG1(F319A) to the chromatin fraction that has been observed after exposure of the cells to KBrO_3_ [70] and thus hSSB1 could act as an additional sensor of damage, guiding OGG1 to the damaged base in specific contexts.

Recently, a high throughput siRNA screening identified 81 proteins whose depletion leads to reduced OGG1 recruitment to chromatin in cells exposed to oxidative stress [72]. Besides proteins already known to be associated with BER, such as UV-DDB1, this work also revealed that several subunits of cohesin and mediator complexes, mostly known for their roles in genome organization [85] and transcriptional regulation [86], contribute to OGG1 recruitment to DNA lesions and facilitate 8-oxoG removal. The involvement of cohesin and mediator complexes in BER, together with their previously known roles in DNA repair of DSB and UV-induced damage, respectively, indicates a major role of these complexes for the maintenance of genomic stability [87,88,89,90].

#### 2.3.2. Potential Role of Post-Translational Modifications in OGG1 Nuclear Dynamics

Several post-translational modifications have been reported for OGG1 and could regulate the affinity of this protein for specific chromatin regions either directly or through cofactors that remain to be identified. Notably, it has been shown that lysine residues in the C-terminal part of OGG1 can be acetylated both in vitro and in vivo by p300. Acetylated OGG1 reduces the affinity of the enzyme for the AP site it generates, increasing its turnover with the consequent enhancement of the 8-oxoG DNA glycosylase activity [91]. Interestingly, histones were the first identified targets of the p300/CBP complex, their acetylation resulting in chromatin relaxation required for transcriptional initiation. The p300/CBP complex is known to acetylate many proteins involved in transcription, DNA replication, and repair, including several BER factors. The DNA glycosylase TDG, responsible for the removal of thymine or uracil mispaired with guanine, commonly associated to CpG islands, is also regulated by p300/CBP. A direct interaction between CBP and TDG was reported and the complex retains both enzymatic activities for DNA repair and transcriptional activation and could thus be considered as a molecular switch to coordinate DNA repair and transcriptional functions [92]. It is worth noting that oxidative stress increases the levels and activity of p300 as well as the levels of acetylated OGG1 [91]. Besides acetylation, it was also reported that several kinases, such as protein kinase C (PKC), cyclin dependent kinase 4 (CDK4), and c-Abl, can phosphorylate OGG1 [93,94]. Phosphorylated OGG1 was shown to be enriched in the chromatin fraction, suggesting that this post-translational modification could modulate the subcellular localization of the enzyme [94].

It is important to keep in mind that post-translational modifications contributing to the arrival or retention of OGG1 at the chromatin could not only alter OGG1 but potentially any other partner. In particular, the post-translational modification of proteins such as cohesin or mediator could provide the signals for attracting OGG1 to regions where DNA is accessible to initiate the scanning process. Further studies are required to have a clearer picture of the post-translational modifications involved in the modulation of OGG1 nuclear distribution upon the induction of 8-oxoG.

### 2.4. Preferential Recruitment of OGG1 to Open Chromatin Regions: A Link with Transcription?

Chromatin is not homogeneously packed within the nucleus and dense regions (heterochromatin) can be distinguished from less compacted ones (euchromatin). Euchromatin is usually associated with active transcription and is thought to be more accessible to proteins.

Upon treatment of cells with oxidizing agents inducing global genome damage, OGG1 is specifically retained in euchromatin regions and clearly excluded from heterochromatin [70]. One possible explanation for the absence of OGG1 in heterochromatic regions could be that their highly compacted structure protects the DNA against oxidation by ROS, reducing the load of 8-oxoG in those regions. However, there is no reason to think that small ROS molecules are unable to reach DNA in heterochromatin [95]. Conversely, the better accessibility of euchromatin could favour those regions as access points for DNA scanning by OGG1. The fact that the mediator and cohesin complexes, which associate with transcriptionally-active genomic area, contribute to OGG1 recruitment to chromatin may also explain the uneven distribution of this glycosylase within the nucleus. The low packing-state and the nucleosome-depletion observed in these euchromatin domains may also facilitate DNA scanning by OGG1 and 8-oxoG clearance [96]. This would be in agreement with the localisation of 8-oxoG that is not randomly distributed in the genome, being detectable at lower levels in genic regions compared to gene deserts [97]. Interestingly, lower levels of 8-oxoG were observed in highly transcribed genes, suggesting a better repair efficiency on those regions [98]. This observation is also consistent with the fact that OGG1 recruitment and assembly of BER complexes occur preferentially in euchromatin regions linked with active transcription [70].

The levels of 8-oxoG in cellular DNA are often used as a biomarker of oxidative stress exposure. Even though 8-oxoG is primarily considered as a base lesion, and its repair by the BER as a mean to avoid mutations, an interesting emerging concept is that due to its high sensitivity to oxidation, guanine in DNA could act as an oxidative stress sensor. Vertebrate genomes are known to be biased in terms of GC content and many promoters of genes coding for stress-response proteins display a high GC content and are particularly sensitive to oxidation [99]. The induction of 8-oxoG at specific sites of the genome, together with its processing by BER, allows the regulation of transcription of certain genes [100,101]. Consistently, recent mapping of the presence of 8-oxoG performed at high resolution showed an enrichment of 8-oxoG in promoters and regulatory regions [99]. Several mechanisms of transcription regulation involving the faithful repair of 8-oxoG at regulatory sequences have been proposed. They include the direct recruitment of transcription factors by BER proteins or repair intermediates, or the stabilization of G-quadruplex structures which in turn drive the recruitment of transcriptional activators [102,103,104]. Furthermore, the use of recently identified OGG1 inhibitors showed that OGG1 activity is required for nuclear factor κB occupancy of its DNA binding sites and proinflammatory gene expression [73]. Those interesting observations place 8-oxoG and OGG1, together with other BER actors, at the crossroad between repair and transcriptional regulation. Further studies are required to better understand this duality and to conciliate the fact that 8-oxoG is at the same time a lesion that hinders genome instability and a signal for the fine-tuning of essential transcription programs.

## 3. Open Questions Regarding the Influence of the Nuclear Environment on OGG1 Functions

As shown in the above sections of this review, the description of the mechanisms of 8-oxoG detection and cleavage by OGG1 along the DNA has now reached a high degree of precision. We have also progressed in the understanding of the roles of co-factors as well as the local chromatin landscape in regulating OGG1 repair activity. There is nevertheless one aspect of this process that remains poorly characterized. That is the impact of the global nuclear environment on the ability of OGG1 to find 8-oxoG lesions within the genome. Indeed, in a context where targets such as for DNA lesions are rare and spread throughout the nucleus, the duration of the diffusive search process might be much longer that the biochemical reaction carried out by the protein on its target. This category of reaction kinetics, often referred to as diffusion-limited, appears widespread among nuclear proteins [105]. Therefore, it is essential to identify the features of the nuclear environment that influence its exploration by OGG1 as well as the strategies developed by this protein to maximize the efficiency of this diffusive searching process.

### 3.1. Does Macromolecular Crowding Facilitate or Impair OGG1 Dynamics within the Nucleus?

Like any nuclear protein searching for a specific target within the nucleus, OGG1 needs to navigate a highly crowded nucleus. An environment is defined as crowded when the macromolecules that are present occupy a significant proportion of the available space. In the nucleus, the main crowding agent is thought to be the chromatin, which occupies 20–40% of the nuclear volume [106]. Because of this high fraction of inaccessible volume, the concentration of other macromolecules is reduced compared to a dilute environment. Besides this so-called volume exclusion effect, crowding is also predicted to dramatically slow down protein diffusion and shift intermolecular reaction equilibria towards the bound state [107].

While in dormant cells crowding seems to play a major role in shutting down key intracellular processes [108], in active cycling cells the contribution of crowding appears less dramatic than predicted by theoretical work. Nevertheless, for tracers of sizes similar to OGG1 a reduction of local concentration of a factor of 1.5 and a slowing-down of diffusion by about 2-fold were reported in dense heterochromatin foci compared to euchromatin [95,109]. Furthermore, chromatin-interacting proteins showing no specificity for histone marks or DNA sequence were shown to be trapped in heterochromatin, probably due to increased binding promoted by crowding [109]. Since these effects are generic, they will also impact OGG1 dynamics and could therefore at least partially explain the differences in behaviour reported for this protein in heterochromatin compared to euchromatin areas [70,72]. Based on in vitro experiments, it was reported that crowding helps to maintain OGG1 nearby the DNA during the scanning of the DNA by consecutive hopping steps [110]. This effect of crowding might actually compensate for the negative impact of the intracellular ionic conditions on OGG1 motion along the DNA [110]. Therefore, by promoting low-dimensionality diffusion phases, crowding could actually accelerate the search phase of repair factors [111]. Nevertheless, theoretical work demonstrates that efficient search by facilitated diffusion requires a trade-off between 1D and 3D diffusion phases [112]. An imbalance in OGG1 facilitated diffusion towards long 1D-sliding phases due to high crowding conditions could ultimately decrease the search efficiency for rare 8-oxoG targets.

### 3.2. How Much Time Does It Take to Find an 8-oxoG Lesion within the Complex Architecture of the Nucleus?

The efficiency of target-search processes has been characterised by two main estimators: the mean first passage time, which estimates the time required for a first encounter between the searcher and its target [113], and the cover time, which estimates the time needed for an exhaustive search in a given area [114]. An interesting finding in the context of DNA repair factors is that target-search strategies minimizing the cover time also optimize the mean first passage time [114], indicating that an efficient and exhaustive scan of the DNA required to avoid leaving some lesions unrepaired is not incompatible with a rapid detection of the lesions. Experimental data assessing these parameters in living cells are unfortunately very sparse. By recording the individual trajectories of DNA ligase and DNA polymerase I in bacteria treated with DNA-damaging agents, Uphoff and colleagues could estimate search times ranging from 10 to 60 s for these two late BER actors [115]. These proteins recognise gaps or nicks along the DNA double-helix, two types of lesions that will have a strong impact on the local conformation of the DNA [116], potentially facilitating the search process. This does not hold true for an 8-oxoG DNA glycosylase, since the cognate lesion has minimal impact on the DNA structure [18] and therefore, the characteristic search time of this protein might be significantly different from the one measured for the ligase or the polymerase.

**Figure 4:**
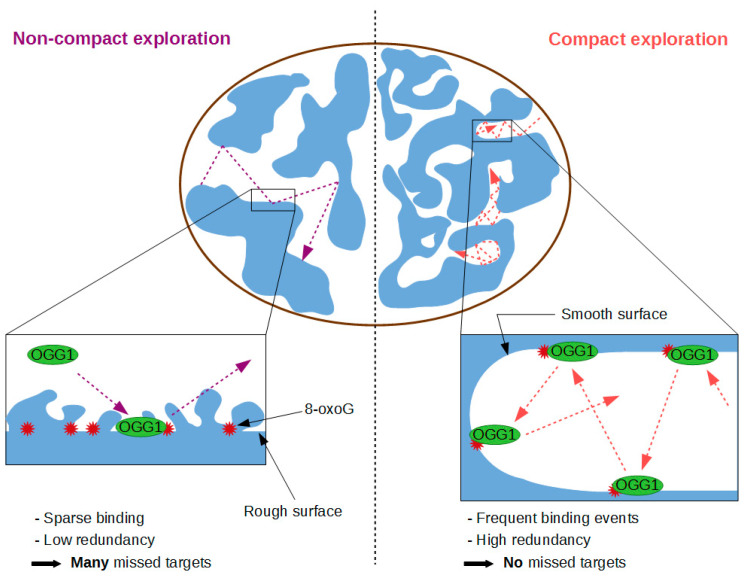
Compact versus non-compact exploration modes in target search.

Besides the time required to find a given target, another more qualitative characteristic of the search process relates to the mode of exploration, which can be either compact or non-compact [117]. In a compact exploration mode, the protein will perform a local exhaustive search, with multiple re-binding events to the same locations. In contrast, non-compact exploration corresponds to a more distant and sparse search which leaves lots of potential binding sites unvisited (Figure 4). Assessing the mode of exploration of nuclear proteins appears crucial since the degree of compactness of the search process is probably related to the biological needs. Analyses of the trajectories of individual transcription factors navigating the nucleus of living mammalian cells have shown that these proteins can actually display opposite exploration strategies [118,119], which were proposed to correspond to different requirements regarding transcription control. For repair proteins, the use of compact exploration would ensure scanning of all DNA and avoid leaving some lesions unrepaired. The published in vitro data suggest that the characteristics of the facilitated diffusion mechanism used by OGG1 leads to high redundancy of the search process, reminiscent of compact exploration processes [61]. Nevertheless, a confirmation of this behaviour in living cells by the tracking of individual fluorescently-tagged OGG1 is still missing.

Importantly, theoretical work indicates that the compactness of a search process, and therefore its redundancy, is not solely governed by the way nuclear proteins diffuse within the nucleus but also strongly depends on the spatial architecture of the chromatin [120,121]. In line with these predictions, experimental work has shown that compact exploration is favoured in heterochromatin domains compared to euchromatin regions [109,122]. It would be interesting to test whether this behaviour is also observed for OGG1, since it would imply more oversampling of the DNA in heterochromatin, which could eventually compensate for the reduced accessibility of these highly crowded areas.

### 3.3. Does Facilitated Diffusion of OGG1 Indeed Occur in the Cell Nucleus?

A very efficient way to speed-up the target-search along the DNA is to use facilitated diffusion for which 3D diffusion phases alternate with exploration at lower dimensionality along the DNA [123]. Nevertheless, to ensure that the search mechanism is not only fast but also highly specific, the proteins must display at least two binding states on the DNA, a transient one responsible for fast scanning and a more stable one associated with the recognition of a specific target [124]. A fast transition between these two binding states appears crucial to overcome the so-called speed/stability paradox [125]. Abundant in vitro data demonstrate that a large spectrum of repair proteins fulfil these different requirements to ensure an efficient clearing of DNA lesions [126,127]. OGG1 is no exception to the rule since its complex scanning process along the DNA described earlier in this review is supposed to allow the rapid and specific detection of the 8-oxoG [128]. Nevertheless, there is a huge gap between our precise understanding of these mechanisms of facilitated diffusion in in vitro reconstituted assays and their actual poor description in a living cell context. Yet, abundant evidence indicates that the high density of the nuclear environment and its ionic conditions may actually significantly impact the efficiency of the facilitated diffusion kinetics observed in a more dilute environment [110,111,129].

In fact, only few papers actually directly show that transcription or repair factors can diffuse along the DNA in living cells [130,131]. Instead, a nearly systematic observation reported in the recent works characterizing the motions of nuclear proteins at the single molecule level is that 3D diffusion alternates with transient nonspecific trapping of the proteins along the DNA [132,133,134]. The characteristic lifetime of these transient interactions appears to be usually extremely short, i.e., below few tens of milliseconds [135,136]. Nevertheless, for some transcription factors, a wide distribution of nonspecific binding times largely overlapping with the characteristic durations of the specific binding events was also reported [137]. A hypothesis to explain these long non-specific trapping periods would be that proteins get confined within small nuclear areas in which they perform consecutive short transient binding that cannot be resolved by the limited resolution of the current single molecule techniques used in living cells [138]. This behaviour could actually correspond to short hopping steps from one DNA chain to another, a process known as intersegmental transfer which was observed in vitro for several transcription and repair factors [127,139,140] (Figure 2). Therefore, even if proper scanning along the DNA may not actually occur within the nuclear context, such local consecutive hopping may transiently lower the dimensionality of the exploration phase, which could be sufficient to speed-up the search process similarly to rigorous facilitated diffusion [141]. OGG1 could actually follow such dynamics since it seems to perform associative 1D sliding only for very short DNA stretches of few nucleotides and rather move along the DNA via hopping [61]. Further analysis of the single-molecule trajectories displayed in living cells by wild-type OGG1 and mutants unable to recognize 8-oxoG should allow a better understanding of how the fine tuning of this multidimensional exploration of the nuclear space promotes the rapid and specific detection of the 8-oxoG lesions along the genome.
